# cAMP Receptor Protein Positively Regulates the Expression of Genes Involved in the Biosynthesis of *Klebsiella oxytoca* Tilivalline Cytotoxin

**DOI:** 10.3389/fmicb.2021.743594

**Published:** 2021-09-30

**Authors:** Diana Rodríguez-Valverde, Nancy León-Montes, Jorge Soria-Bustos, Jessica Martínez-Cruz, Ricardo González-Ugalde, Sandra Rivera-Gutiérrez, Jorge A. González-y-Merchand, Roberto Rosales-Reyes, Lázaro García-Morales, Hidetada Hirakawa, James G. Fox, Jorge A. Girón, Miguel A. De la Cruz, Miguel A. Ares

**Affiliations:** ^1^Unidad de Investigación Médica en Enfermedades Infecciosas y Parasitarias, Hospital de Pediatría, Centro Médico Nacional Siglo XXI, Instituto Mexicano del Seguro Social, Mexico City, Mexico; ^2^Departamento de Microbiología, Escuela Nacional de Ciencias Biológicas, Instituto Politécnico Nacional, Mexico City, Mexico; ^3^Unidad de Medicina Experimental, Facultad de Medicina, Universidad Nacional Autónoma de México, Mexico City, Mexico; ^4^Departamento de Biomedicina Molecular, Centro de Investigación y de Estudios Avanzados del Instituto Politécnico Nacional, Mexico City, Mexico; ^5^Department of Bacteriology, Gunma University Graduate School of Medicine, Maebashi, Japan; ^6^Division of Comparative Medicine, Massachusetts Institute of Technology, Cambridge, MA, United States; ^7^Centro de Detección Biomolecular, Benemérita Universidad Autónoma de Puebla, Puebla, Mexico

**Keywords:** CRP, tilivalline cytotoxin, *aroX*, *npsA*, *Klebsiella oxytoca*

## Abstract

*Klebsiella oxytoca* is a resident of the human gut. However, certain *K. oxytoca* toxigenic strains exist that secrete the nonribosomal peptide tilivalline (TV) cytotoxin. TV is a pyrrolobenzodiazepine that causes antibiotic-associated hemorrhagic colitis (AAHC). The biosynthesis of TV is driven by enzymes encoded by the *aroX* and NRPS operons. In this study, we determined the effect of environmental signals such as carbon sources, osmolarity, and divalent cations on the transcription of both TV biosynthetic operons. Gene expression was enhanced when bacteria were cultivated in tryptone lactose broth. Glucose, high osmolarity, and depletion of calcium and magnesium diminished gene expression, whereas glycerol increased transcription of both TV biosynthetic operons. The cAMP receptor protein (CRP) is a major transcriptional regulator in bacteria that plays a key role in metabolic regulation. To investigate the role of CRP on the cytotoxicity of *K. oxytoca*, we compared levels of expression of TV biosynthetic operons and synthesis of TV in wild-type strain MIT 09-7231 and a Δ*crp* isogenic mutant. In summary, we found that CRP directly activates the transcription of the *aroX* and NRPS operons and that the absence of CRP reduced cytotoxicity of *K. oxytoca* on HeLa cells, due to a significant reduction in TV production. This study highlights the importance of the CRP protein in the regulation of virulence genes in enteric bacteria and broadens our knowledge on the regulatory mechanisms of the TV cytotoxin.

## Introduction

The human gut microbiota is a complex community of microbial species that plays a fundamental role in the health and functioning of the human digestive tract. The homeostasis of this community provides protection against pathogens (Belkaid and Harrison, 2017; Lin et al., 2021). However, the use of antibiotics can break up this ecosystem and cause dysbiosis. Intestinal dysbiosis is defined as a cutback of beneficial commensal bacteria and development of damaging commensal bacteria termed opportunistic pathogens or pathobionts (Nagao-Kitamoto and Kamada, 2017; Kitamoto et al., 2020; Wei et al., 2021). The dysbiotic gut microbiota can trigger the initiation of gastrointestinal diseases including the antibiotic-associated hemorrhagic colitis (AAHC). Hospitalized patients who receive treatment with antibiotics may develop AAHC due to the pathobiont *Klebsiella oxytoca,* a Gram-negative bacterium that resides in the human gut. Toxigenic *K. oxytoca* strains carry a gene cluster that codes for proteins that synthesize a cytotoxin known as tilivalline (TV), which is largely responsible for this disease (Dornisch et al., 2017). Unlike other toxins, TV is not a protein, but a pentacyclic pyrrolobenzodiazepine metabolite. The cytotoxin biosynthetic gene cluster is a part of a pathogenicity island (PAI) and is organized in two operons termed *aroX* and NRPS. The *aroX* operon is a 6.1-kbp region encoding five genes: *aroX*, *dhbX*, *icmX*, *adsX*, and *hmoX*. The NRPS operon is a 6.2-kbp region encoding three genes: *npsA*, *thdA*, and *npsB* (Schneditz et al., 2014; Dornisch et al., 2017; Tse et al., 2017). TV disrupts cell cycle progression due to the enhancement of nucleation and elongation of tubulin polymerization (Unterhauser et al., 2019). Furthermore, TV induces epithelial apoptosis and changes the expression and localization of the tight junction protein claudin-1. Consequently, the intestinal barrier function is impaired (Schneditz et al., 2014; Hering et al., 2019).

The regulation of the expression of the *aroX* and NRPS operons has not been studied. Pathogenic bacteria possess a myriad of transcription factors that control their virulence (Crofts et al., 2018; Huang et al., 2019; King et al., 2020; Lee et al., 2020; Ramamurthy et al., 2020; Wójcicki et al., 2021). The cAMP receptor protein (CRP) is one of the most important global regulators controlling the expression of many genes in bacteria. It has been associated with the regulation of virulence factors, pathogenicity islands, and enzymes involved in the metabolism of various enterobacterial species such as *Klebsiella pneumoniae*, enterotoxigenic *Escherichia coli*, *Salmonella enterica* serovar Typhimurium, and *Pseudomonas aeruginosa* (De la Cruz et al., 2016, 2017; Xue et al., 2016; Berry et al., 2018; El Mouali et al., 2018; Ares et al., 2019). CRP consists of a homodimer, and its function depends on its binding to cAMP. When such interaction occurs, the conformation of CRP changes and this allows it to bind to the promoters on DNA in order to regulate transcriptional expression (Lindemose et al., 2014; Gunasekara et al., 2015; Xu et al., 2021). There are no studies on the role of the global regulator CRP in *K. oxytoca*. Nevertheless, in its close relative *K*. *pneumoniae,* CRP is a negative regulator of the capsular polysaccharide and a positive regulator of type 3 fimbriae (Ou et al., 2017; Panjaitan et al., 2019). In *E. coli,* CRP has more than 260 binding sites (Salgado, 2004) and controls the transcription of genes that code for proteins involved in a wide range of cellular processes, including its well-known role in the regulation of the *lac* operon, biofilm formation, iron uptake, antibiotic multidrug resistance, quorum sensing, shikimate pathway, and oxidative stress resistance (Jiang and Zhang, 2016; Uppal and Jawali, 2016; Ritzert et al., 2019; Liu et al., 2020b; Kumar et al., 2021). Additionally, CRP is involved in catabolite repression and metabolism of carbon sources. For example, in the absence of some rapidly metabolizable carbon sources such as glucose, CRP activates adenylate cyclase, which leads to an increase in enzyme production involved in the use of alternative carbon sources, such as lactose (Salgado, 2004; Geng and Jiang, 2015; Liu et al., 2020a). Indeed, it was previously reported that culturing a cytotoxin-producing *K*. *oxytoca* strain in a lactose-containing medium increases the production of TV and consequently the cytopathic effect on epithelial cells (Tse et al., 2017).

In this study, we sought to investigate the role of CRP in the transcriptional control of the enzymes encoded by the *aroX* and NRPS operons in the cytotoxin-producing *K*. *oxytoca* strain MIT 09-7231 (Darby et al., 2014). In addition, we evaluated the expression of *aroX* and NRPS operons in different environmental growth conditions including utilization of various carbon sources. Furthermore, we investigated the role of CRP protein in the transcriptional regulation of *aroX* and NRPS operons. The regulatory effect of CRP in production of TV cytotoxin was also analyzed by cytotoxicity assays on HeLa cells. To the best of our knowledge, this is the first study addressing the transcriptional regulation of the TV biosynthetic gene cluster, in which CRP acts as an activator.

## Materials and Methods

### Bacterial Strains and Growth Conditions

*Klebsiella oxytoca* strains and bacterial constructs used in this study are listed in Table 1. *K. oxytoca* MIT 09-7231 was used as the prototypic toxigenic strain and for construction of the isogenic Δ*crp* mutant (Darby et al., 2014). To determine gene expression of *aroX* and NRPS operons, different liquid bacteriological media were used: lysogeny broth (LB), tryptone soy broth (TSB), tryptone lactose broth (TLB), and Dulbecco’s modified Eagle’s medium (DMEM) with high glucose (4.5g/l). The transcription of *aroX* and *npsA* genes was analyzed under different environmental conditions in TLB medium at 37°C with shaking, and samples were harvested when an OD_600nm_ of 1.6 was reached for RNA extraction. TLB medium was prepared as previously described [17g/l tryptone, 10g/l lactose, and 2.5g/l dipotassium hydrogen phosphate (Tse et al., 2017)]. TLB medium was supplemented with 0.2% glucose, 0.2% glycerol, 0.3M NaCl, 1.0mM EDTA, 5.0mM CaCl_2_, or 5.0mM MgCl_2_. In order to examine the effect of lactose on the transcription of *aroX* and *npsA* genes, tryptone broth (TB) was used and gene expression results were compared with those obtained from cultures in TLB medium. Antibiotics [200μg/ml (ampicillin), 50μg/ml (kanamycin), or 10μg/ml (tetracycline)] were added to culture media when necessary.

**Table 1:** Bacterial strains and plasmids used in this study.

Strain or plasmid	Description	References
Strains		
*K*. *oxytoca* WT	Wild-type *K*. *oxytoca* strain MIT 09-7231	Darby et al., 2014
*K. oxytoca* Δ*crp*	*K. oxytoca* Δ*crp*::FRT	This study
*K. oxytoca* Δ*npsA*	*K. oxytoca* Δ*npsA*::FRT	This study
*E. coli* BL21(DE3)	F^−^*omp*T *hsd*S_B_ (r_B_^−^, m_B_^−^) *gal dcm* (DE3)	Invitrogen
Plasmids		
pTrc99Acrp	*crp* expression plasmid; Ap^R^	Kurabayashi et al., 2017
pTrc99K-CRP	*crp* expression plasmid; Km^R^	This study
pQE80crp	N-terminal His_6_-Crp overexpression plasmid; Ap^R^	Kurabayashi et al., 2017
pKD119	pINT-ts derivative containing the λ Red recombinase system under an arabinose-inducible promoter, Tc^R^	Datsenko and Wanner, 2000
pKD4	pANTsy derivative template plasmid containing the kanamycin cassette for λ Red recombination, Ap^R^	Datsenko and Wanner, 2000
pCP20	Plasmid that shows temperature-sensitive replication and thermal induction of FLP synthesis, Ap^R^, Cm^R^	Datsenko and Wanner, 2000

### Construction of Isogenic Mutants

*K. oxytoca* MIT 09-7231 was targeted for mutagenesis of *crp* and *npsA* genes by using the lambda-Red recombinase system (Datsenko and Wanner, 2000). Briefly, PCR fragments containing *crp* or *npsA* sequences flanking a kanamycin resistance gene were obtained by using gene-specific primer pairs (Table 2). Each purified PCR product was electroporated independently into competent *K. oxytoca* carrying the lambda-Red recombinase helper plasmid pKD119, whose expression was induced by the addition of L-(+)-arabinose (Sigma) at a final concentration of 1%. The mutations were confirmed by PCR and sequencing. Subsequently, the FRT-flanked kanamycin cassettes were excised from both Δ*crp* and Δ*npsA* mutant strains after transformation with pCP20 plasmid, as previously described (Datsenko and Wanner, 2000).

**Table 2:** Primers used in this study.

Primer	Sequence (5"- 3")	Target gene
For Gene Deletion		
crp-H1P1	TATAACAGAGGATAACCGCGCATGGTGCTTGGCAAACCGCAAACATGTAGGCTGGAGCTGCTTCG	*crp*
crp-H2P2	GCAATACGCCGTTTTACCGACTTAACGGGTACCGTAGACGACGATCATATGAATATCCTCCTTAG	
npsA-H1P1	CTAATTCTCCAGGAGAGAGTGATGACGCATTCAGCATATGTCTATTGTAGGCTGGAGCTGCTTCG	*npsA*
npsA-H2P2	GTTGCTCAACGTTGTCCATATTTACACCTGCTCCAGTAAAGAATTCATATGAATATCCTCCTTAG	
For Site-Directed Mutagenesis		
aroX-npsA-CRPBox-F	TGCCGCCAGCTTACCACAGGATGCCCTCGGGCAAACACCGCAAAA	*aroX*-*npsA*
aroX-npsA-CRPBox-R	TTTTGCGGTGTTTGCCCGAGGGCATCCTGTGGTAAGCTGGCGGCA	
For Mutant Characterization		
crp-MC-F	CGGCACCCGGAGATAGCTTA	*crp*
crp-MC-R	AGGGGAAAACAAAAACGGCG	
npsA-MC-F	TTTGCGGTGTTTTCTTAGAAGCA	*npsA*
npsA-MC-R	CGGGTTAATCGCCTCTGAATG	
FOR qPCR		
aroX-F	TGTTGCCTGCAAGATTGACG	*aroX*
aroX-R	ATGTGTGAACGGCCAAAACG	
dhbX-F	ATGCGGCCAATCTGATGATG	*dhbX*
dhbX-R	AGCCCCAGAGCATAGGTAAATG	
icmx-F	TGATTGTCTGCGGCGTTTAC	*icmX*
icmX-R	GCTAGACGATGCTTTTCTTCGG	
adsX-F	TGCACATTGAACGGCAAGAC	*adsX*
adsX-R	ATCGAAGTGCAGGTTTCGTG	
hmoX-F	TCGCATGCCAAAGATTTCGC	*hmoX*
hmoX-R	ATGAGCTTGACGCGTTCAAC	
npsA-F	AAATACGTGGCTTCCGCATC	*npsA*
npsA-R	TCCTGCGTGACATAACAAGC	
thdA-F	TGGACAACGTTGAGCAACAG	*thdA*
thdA-R	TGCTTACCATTGACGCCAAC	
npsB-F	TGAGCATTTGCAGCTGGTTC	*npsB*
npsB-R	ATGCGTGGCAACTTTGTGTG	
crp-F	TGCTGAACCTGGCAAAACAG	*crp*
crp-R	ATTTTCAGGATGCGGCCAAC	
rrsH-F	CAGGGGTTTGGTCAGACACA	*rrsH*
rrsH-R	GTTAGCCGGTGCTTCTTCTG	
For EMSA		
aroX-npsA-F	TCTCTCACTCGAAATTTAACAGGT	*aroX*-*npsA*
aroX-npsA-R	TCTCTCCTGGAGAATTAGGAACG	
estA2-F	CCAGAGGCGGTCGAACTC	*estA2*
estA2-R	ATTACCTCCGAAACACGTCGT	
eltA-F	CCAGCGATAAAGTCTGTAAATACGG	*eltA*
eltA-R	TATCATACAAGAAGACAATCCGGA	

*The sequence corresponding to the template plasmid pKD4 is underlined*.

### Construction of ΔCRP-Box Mutant Probe by Site-Directed Mutagenesis

A fragment with targeted mutations in the putative CRP-Box of intergenic regulatory region of the divergent *aroX* and *npsA* genes was generated using overlapping PCR (Ho et al., 1989) with specific primers (Table 2). Two fragments were generated separately in a first round of PCR: one with the 5΄ half and other with the 3΄ half of the intergenic regulatory region of *aroX* and *npsA* genes including the overlapping mutated region. Subsequently, the two fragments were mixed and amplified in a second round of PCR. DNA sequencing was carried out to verify the introduction of the point mutations.

### Construction of pTrc99K-CRP Plasmid

The pTrc99K-CRP plasmid was constructed for complementation experiments by subcloning the *crp* gene from the pTrc99Acrp plasmid (Kurabayashi et al., 2017). The pTrc99Acrp vector was digested with NcoI and BamHI, and the fragment corresponding to the *crp* gene was then purified and ligated into pTrc99K previously digested with the same restriction enzymes. The identity of the insert was confirmed by DNA sequencing.

### RNA Extraction and Quantitative RT-PCR

Total RNA was extracted from bacteria grown under different culture conditions using the hot phenol method (Jahn et al., 2008; Ares, 2012), with some modifications. Briefly, after the lysate was obtained, an equal volume of phenol-saturated water was added, mixed, and incubated at 65°C for 5min. The samples were chilled on ice and centrifuged at 19,000 × *g* for 10min at 4°C. The aqueous layer was transferred to a microtube, RNA was precipitated with cold ethanol, and it was incubated at −70°C overnight.

The RNA was pelleted by centrifugation at 19,000 x *g* for 10min at 4°C. Pellets were washed with cold 70% ethanol and centrifuged at 19,000 × *g* for 5min at 4°C. After careful removal of the ethanol, the pellets were air-dried for 15min in a Centrifugal Vacuum Concentrator 5,301 (Eppendorf). The pellets were resuspended in DEPC-treated water. Purification of RNA was performed using the TURBO DNA-free kit (Ambion, Inc.). Quality of RNA was assessed using the NanoDrop ONE (Thermo Scientific) and with a bleach denaturing 1.5% agarose gel, as previously described (Aranda et al., 2012). cDNA was synthesized using 1 μg of RNA, 5pmol/μl of random hexamer primers, and 200U/μl of RevertAid M-MulV-RT (Reverse transcriptase of Moloney Murine Leukemia Virus; Thermo Scientific).

Quantitative real-time PCR was performed in a LightCycler 480 instrument (Roche) to quantify the gene expression levels. Specific primers (Table 2) were designed using the Primer3Plus software^1^ (Untergasser et al., 2007). For LightCycler reactions, a master mix of the following components was prepared: 2.0 μl of PCR-grade water, 0.5 μl (10 μM) of forward primer, 0.5 μl (10 μM) of reverse primer, 5 μl of 2x Master Mix, and 2.5 μl of cDNA (~50ng). A multiwell plate containing all samples was loaded into the LightCycler 480 instrument. Amplification was performed in triplicate wells for each sample analyzed from three independent experiments. In each set of reactions, 16S rRNA (*rrsH*) was used as a reference gene for normalization of the cDNA amount. Real-time PCR analysis was performed using the following optimized assay conditions: (1) denaturation program (95°C for 10min); amplification and quantification programs were repeated for 45cycles (95°C for 10s, 59°C for 10s, 72°C for 10s with a single fluorescence measurement), (2) melting curve program (95°C for 10s, 65°C for 1min with continuous fluorescence measurement at 97°C), and (3) a cooling step at 40°C for 10s. The absence of contaminating DNA was tested by the lack of amplification products after 45 qPCR cycles using RNA as template. Control reactions with no RNA template and with no reverse transcriptase enzyme were run in all experiments. The relative gene expression was calculated using the 2^-ΔΔCt^ method (Livak and Schmittgen, 2001; Schmittgen and Livak, 2008). To ensure that 16S rRNA (*rrsH*) was an optimal reference gene for normalization of qPCR, absolute quantification was performed by obtaining a standard curve according to 10-fold dilutions of *K. oxytoca* 09–7231 chromosomal DNA (10^3^, 10^4^, 10^5^, 10^6^, and 10^7^ theoretical copies). Ct values were interpolated to standard curve to obtain gene expression (gene copies per μg RNA). Expression of 16S rRNA (*rrsH*) gene remained unaffected in all conditions tested (Supplementary Figure S1).

### Protein Purification

The His_6_-CRP expression plasmid pQE80crp (Kurabayashi et al., 2017) was electroporated into competent *E. coli* BL21 (DE3). Bacteria containing recombinant plasmid were grown at 37°C to an OD_600nm_ of 0.5 in LB; 1.0mM IPTG was then added and cultured for 3h. Cells were then pelleted by centrifugation and resuspended in urea buffer (8M urea, 100mM Na_2_HPO_4_, and 10mM Tris-HCl, pH 8.0) and lysed by sonication. The lysate was centrifuged, and the supernatant was filtered through Ni-NTA agarose column (Qiagen) pre-equilibrated with urea buffer. After washing with buffer containing 50mM imidazole (200ml), the protein was eluted with 500mM imidazole (10ml). Fractions were analyzed by SDS-PAGE and Coomassie blue staining. Protein concentration was determined by the Bradford procedure. Aliquots of the purified protein were stored at −70°C.

### Electrophoretic Mobility Shift Assays

To evaluate CRP binding to the promotor sequence, a 448-bp DNA probe containing the intergenic regulatory region of the divergent *aroX* and *npsA* genes was used. In addition, the probe ΔCRP-Box containing the mutation in the putative CRP-binding site on the intergenic regulatory region of the divergent *aroX* and *npsA* genes was employed. DNA probes from the regulatory region of the enterotoxigenic *E. coli estA2* and *eltA* genes were used as positive and negative controls (Haycocks et al., 2015). The binding reaction was performed with 100ng of DNA probes and increasing concentrations of purified His_6_-CRP with or without 200μM cAMP (Hirakawa et al., 2020) in a 20μl reaction mixture containing H/S 10X gel-shift binding buffer (400mM HEPES, 80mM MgCl_2_, 500mM KCl, 10mM DTT, 0.5% NP40, and 1mg/ml BSA, De la Cruz et al., 2007). Samples were incubated for 20min at room temperature and then separated by electrophoresis in 6% non-denaturing polyacrylamide gels using 0.5X Tris-borate-EDTA buffer at 4°C. The DNA bands were stained with ethidium bromide and visualized under UV light.

### Cytotoxicity Assays

HeLa cell line (ATCC CCL-2) was used to determine cytotoxic activity as previously described (Darby et al., 2014). Approximately 1×10^6^ cells were suspended in 900μl of DMEM high glucose (4.5g/l; Invitrogen) with 10% FBS (Gibco) and seeded into 24-well cell culture plates (Costar). To investigate cytotoxin production in the *K. oxytoca* strains (wild type, Δ*crp*, Δ*crp* pTrc99K-CRP, and Δ*npsA*), 100μl of bacterial supernatants was filtered through a PVDF 0.22-μm sterile Millex-GV filter (Merck Millipore) and added to the wells containing HeLa cells. After 48h of incubation at 37°C under a 5% CO_2_ atmosphere, the cells were visualized using a Nikon TE300 inverted microscope at 10X magnification. Cytotoxin production was defined as >50% cell rounding and detachment and<50% confluency, as compared to the negative control samples [(TLB medium only or supernatant of the non-toxigenic *K. oxytoca* Δ*npsA* strain (Schneditz et al., 2014; Dornisch et al., 2017; Tse et al., 2017)]. Negative control samples had a monolayer with minimal cell rounding or detachment and>80% confluency.

The LDH Cytotoxicity Assay Kit (Invitrogen) was used according to the manufacturer’s instructions to measure lactate dehydrogenase (LDH) released from HeLa cells after damage of plasma membrane integrity. 1×10^4^ HeLa cells were cultivated in 100μl DMEM high glucose (4.5g/l; Invitrogen) with 10% FBS (Gibco), seeded into 96-well flat-bottom culture plates (Costar), and incubated at 37°C under a 5% CO_2_ atmosphere for 48h. Subsequently, the medium was removed, and the cells washed with PBS. Then, 10μl of negative control (PBS), culture medium control (TLB), positive control (lysis buffer), and filtered bacterial supernatants (wild type, Δ*crp*, Δ*crp* pTrc99K-CRP, and Δ*npsA*) as described before was added to DMEM medium without FBS for 48h. After treatment, an aliquot of 50μl each sample medium was transferred to a new 96-well plate, and kit solutions were added into each well. The absorbance was measured at 490nm and 680nm with a spectrophotometer (Multiskan Ascent, Thermo Scientific). All samples were tested in triplicate on three independent biological replicates, and the mean results were expressed as LDH cytotoxicity by subtracting the 680nm absorbance background value from the 490nm absorbance value.

### Statistical Analysis

Statistical analysis was performed using Prism 7.0 (GraphPad Software, Inc., San Diego, CA, United States). Data represent the mean±standard deviation (SD). The mean differences were determined using one-way ANOVA followed by Tukey’s comparison test. Values of *p*<0.05 were considered statistically significant.

## Results

### Expression of *aroX* and NRPS Operons Is Enhanced by Growth in TLB Medium

**Figure 1:**
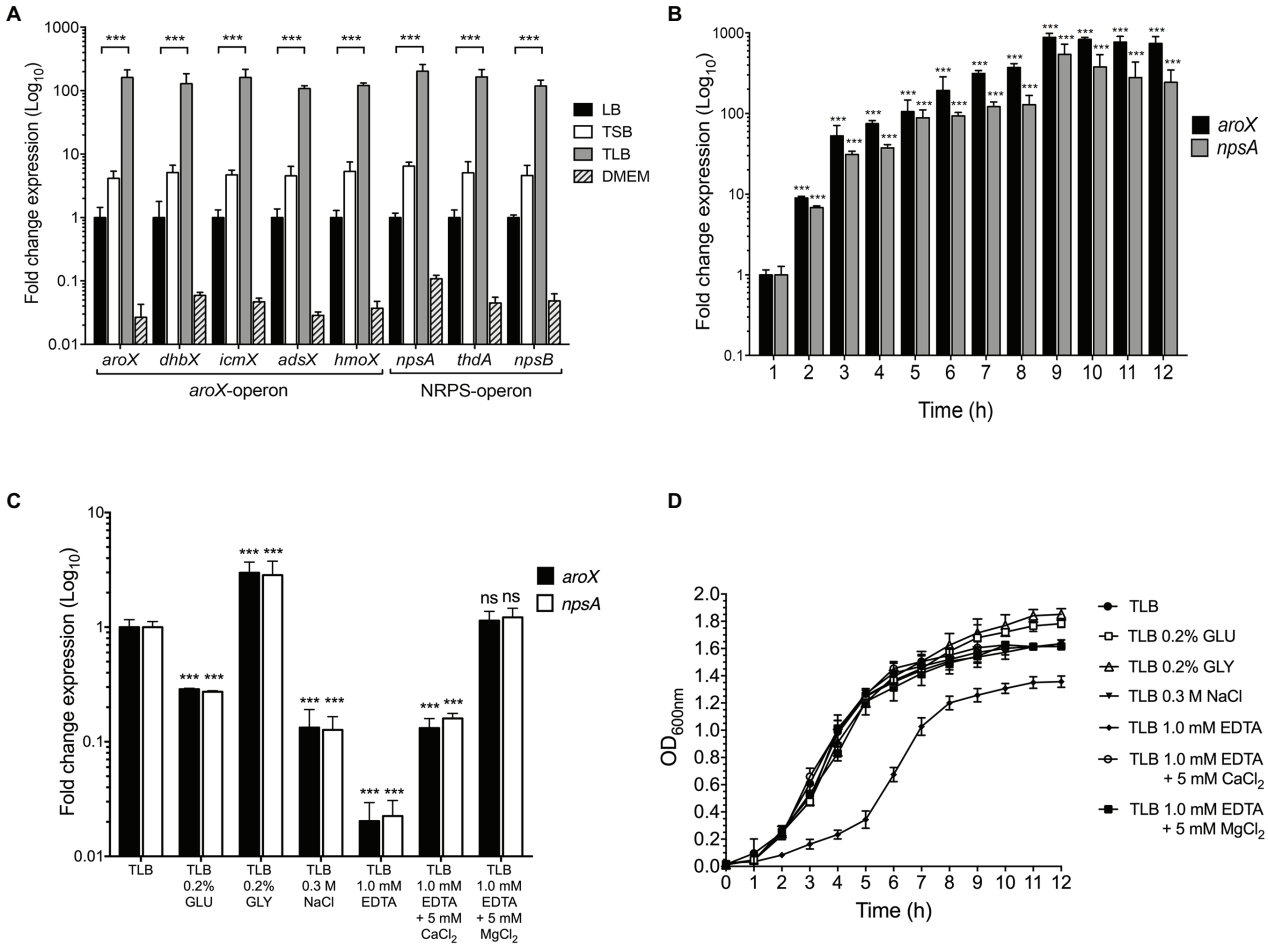
Effect of environmental cues on *aroX* and NRPS operons expression. **(A)** Fold change expression detected by RT-qPCR of *aroX* and NRPS operons of *K. oxytoca* compared to LB. Bacterial cultures were grown at 37°C for 9h in different culture media: lysogenic broth (LB), tryptone soy broth (TSB), tryptone lactose broth (TLB), and Dulbecco’s modified Eagle’s medium (DMEM) with high glucose (4.5g/l). **(B)** Transcription of *aroX* and *npsA* genes during growth phases. **(C)** Transcription of *aroX* and *npsA* genes at stationary phase (OD_600nm_=1.6) at 37°C under different environmental conditions determined by RT-qPCR. **(D)** Growth curves of wild-type *K. oxytoca* in TLB medium at 37°C for 12h supplemented with glucose (GLU, 0.2%), glycerol (GLY, 0.2%), sodium chloride (NaCl, 0.3M), and ethylenediaminetetraacetic acid (EDTA, 1.0mM, supplemented or not supplemented with 5.0mM CaCl_2_/MgCl_2_). 16S rRNA was used as a reference gene for normalization of expression. These graphs represent the mean of three independent experiments performed in triplicate with standard deviations. Statistically significant with respect to bacteria grown in LB medium (A) or with respect to bacteria grown after 1h post-inoculation in TLB medium (B) or with respect to bacteria grown in non-supplemented TLB medium (C): ^**^p<0.001. All values of *p* were calculated using one-way ANOVA and Tukey’s comparison test.

To determine the optimal conditions of expression of the *aroX* and NRPS operons of *K. oxytoca* MIT 09-7231, the bacteria were cultivated in different culture media, such as LB, TSB, TLB, and DMEM transcription analyzed by RT-qPCR. The conventional LB medium was used as a reference to determine the basic levels of expression of genes encoded by the *aroX* and NRPS operons. The lowest levels of transcription of the *aroX* and NRPS operons occurred when the bacteria were cultivated in DMEM; this was rather surprising since expression of most virulence factors of pathogenic *E. coli* strains occurs upon growth in DMEM (Leverton and Kaper, 2005; Platenkamp and Mellies, 2018). In comparison with the growth of MIT 09-7231 in LB, the expression of *aroX* and NRPS operons of MIT 09-7231 cultivated in TSB and TLB media enhanced ~5- and~150-fold, respectively (Figure 1A). Unlike TSB medium, TLB contains lactose instead of soy. The expression levels of TV genes were very similar in the different culture media, supporting the notion that they are genetically organized in operons. Since *aroX* and *npsA* are the first genes of the *aroX* and NRPS operons, respectively, only the expression of these two genes was evaluated throughout the study. When *K. oxytoca* MIT 09-7231 was grown in TLB at 37°C for 12h, the highest levels of expression of *aroX* and *npsA* genes occurred at 9h and were maintained for 12h, which corresponds to stationary growth phase (Figure 1B). Our data indicate that growth in TLB medium, which contains lactose, favors the expression of *K. oxytoca aroX* and NRPS operons during stationary growth phase.

### Expression of *aroX* and *npsA* Genes Is Differentially Regulated by Nutritional and Environmental Factors

The influence of nutritional factors in the expression of genes involved in TV biosynthesis was quantified by RT-qPCR when *K. oxytoca* was grown in TLB supplemented with 0.2% glucose, 0.2% glycerol, 0.3M sodium chloride, and 1.0mM EDTA. The expression of *aroX* and *npsA* genes was repressed (~4-fold) and activated (~3-fold) by glucose and glycerol, respectively (Figure 1C). In high osmolarity (0.3M NaCl), the transcription of *aroX* and *npsA* decreased ~8-fold (Figure 1C). Transcription of these genes required divalent cations because the addition of EDTA dramatically diminished their expression (~50-fold), and this effect was partially and fully reverted by the addition of CaCl_2_ and MgCl_2_, respectively (Figure 1C). Of note, depletion of divalent cations by the addition of EDTA to the culture medium affected negatively bacterial growth (Figure 1D). This effect was reversed by the addition of CaCl_2_ and MgCl_2_ to TLB containing EDTA (Figure 1D). The data indicate that glucose, high osmolarity, and depletion of divalent cations from the growth medium repress *aroX* and *npsA* genes.

### CRP Activates the Expression of *aroX* and *npsA* Genes

**Figure 2:**
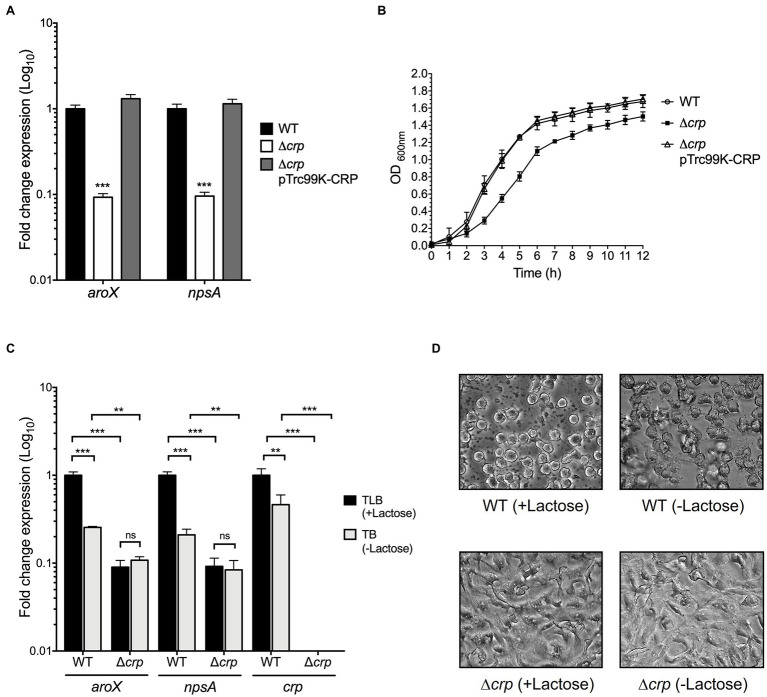
Regulatory activity of CRP in expression of *aroX* and *npsA* genes. **(A)** Determination of transcriptional expression by RT-qPCR of *aroX* and *npsA* genes of wild-type *K. oxytoca*, Δ*crp*, and Δ*crp* pTrc99K-CRP in TLB medium at stationary phase (OD_600nm_=1.6) at 37°C. **(B)** Growth curves of wild-type *K. oxytoca*, Δ*crp*, and Δ*crp* pTrc99K-CRP, in TLB medium at 37°C. Bacterial cultures were grown for 12h. **(C)** Transcription of *aroX*, *npsA*, and *crp* genes at stationary phase (OD_600nm_=1.6) at 37°C determined by RT-qPCR in TLB (medium with lactose), and TB (medium without lactose) at 37°C for 12h. These graphs represent the mean of three independent experiments performed in triplicate with standard deviations. **(D)** HeLa cell culture inoculated with supernatants recovered from wild-type and Δ*crp* strains grown in TLB (medium with lactose) and TB (medium without lactose). Results represent the mean of three independent experiments performed in triplicate with standard deviations. Statistically significant with respect to wild-type strain (A) or with respect to bacteria grown in TLB medium (B): ^**^p<0.01; ^***^p<0.001. All Values of *p* were calculated using one-way ANOVA and Tukey’s comparison test.

As described above, carbon sources such as lactose, glucose, and glycerol affect the transcription of *aroX* and *npsA* genes. CRP is a global regulator that senses the fluctuations of carbon sources and controls the transcription of some enzymes involved in metabolite biosynthesis (Bai et al., 2011; Gao et al., 2012; Soberón-Chávez et al., 2017; Jeong et al., 2021). Hence, we sought to investigate the role of CRP in the regulation of *aroX* and *npsA* genes. Growth rates and expression of these genes in the wild type and its derivative ∆*crp* isogenic mutant were compared after growth in TLB at stationary phase (OD_600nm_=1.6) by RT-qPCR. In the absence of CRP, growth was significantly attenuated and expression levels of both *aroX* and *npsA* genes were diminished ~10-fold. Both, growth rate and expression were reversed by the complementation of this mutant with the pTrc99K-CRP plasmid (Figures 2A,B). These results indicate that CRP positively regulates expression of *aroX* and *npsA* genes.

### Effect of Lactose on CRP-Mediated *aroX* and *npsA* Expression

We wanted to know whether the regulation exerted by lactose on TV genes implicated CRP. Thus, we quantified the transcription of *aroX* and *npsA* genes in the wild-type and ∆*crp* mutant strains growing in TLB (medium with lactose) and TB (tryptone broth), which is TLB without lactose. Growth of the wild-type strain in the absence of the lactose decreased TV gene transcription by ~5-fold (Figure 2C). This effect was CRP-dependent because transcription of *aroX* and *npsA* was not altered in the ∆*crp* mutant strain (Figure 2C). As an expression control of a lactose-regulated gene, we quantified the transcription of *crp* in the wild-type growing in the presence of lactose and found ~2-fold increase in expression. We hypothesized that lactose is involved in the CRP-dependent *aroX* and *npsA* transcription; thus, we analyzed TV-mediated cytotoxicity on HeLa epithelial cells using the supernatants of the wild-type and ∆*crp* mutant cultures grown in the presence and absence of lactose (Figure 2D). The supernatant recovered of the wild-type strain grown in TLB (with lactose) presented a higher cytotoxic effect on HeLa cells than the supernatant obtained from the wild-type strain grown in TB (without lactose). The supernatants from the ∆*crp* mutant grown with/without lactose caused a slight cytotoxic effect on HeLa cells (Figure 2D). These data support our hypothesis that lactose induces CRP-mediated *aroX* and *npsA* genes transcription and consequently triggers TV-mediated cytotoxicity on HeLa epithelial cells.

### CRP Binds to the Intergenic Region of *aroX* and *npsA* Genes

**Figure 3:**
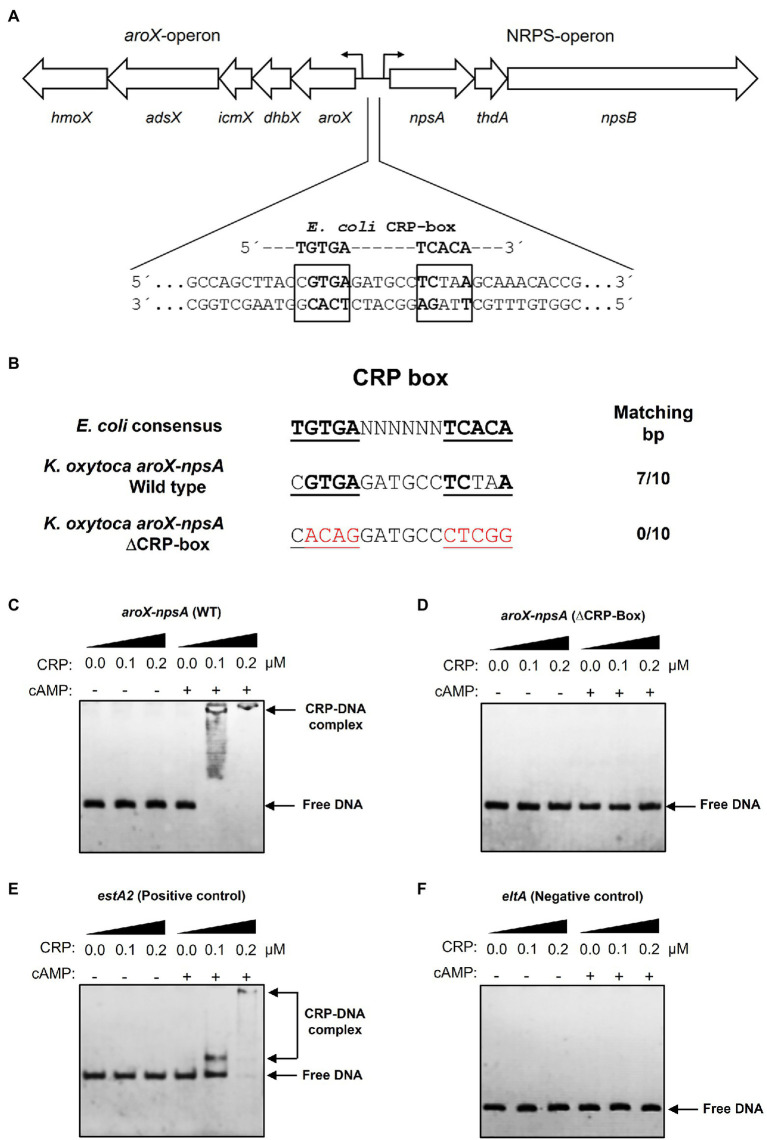
Electrophoretic mobility shift assay showing binding of CRP-cAMP to the intergenic region of *aroX* and *npsA* genes. **(A)** Genetic organization of the *aroX* and NRPS operons and putative CRP-binding sites located in the intergenic regulatory region. The putative CRP-binding site is indicated with bold and boxed letters. **(B)** The intergenic region of *K. oxytoca aroX* and *npsA* genes contains a CRP-Box similar to the CRP-binding consensus sequence found in *E. coli*. An altered CRP-Box was generated to determine CRP binding. Bases matching the consensus sequence are bold, and mutated bases are shown in red. EMSA experiments were conducted to determine the binding of purified recombinant His_6_-CRP protein to the corresponding DNA probe from the wild-type intergenic region of *aroX* and *npsA* genes **(C)** and from the intergenic region of *aroX* and *npsA* containing the mutation in the putative CRP-Box **(D).** DNA probes from the *estA2* **(E)** and *eltA* **(F)** regulatory regions were used as positive and negative controls, respectively. 100ng of DNA probe of each regulatory region was mixed and incubated with increasing concentrations (μM) of purified recombinant His_6_-CRP protein (CRP) in the presence or absence of 200μM of cAMP. Free DNA and CRP-DNA complex stained with ethidium bromide are indicated.

Sequence analysis of the intergenic region of *aroX* and *npsA* genes identified a putative CRP-binding site (Figure 3A). This sequence (CGTGA-N_6_-TCTAA) shared 7 of 10bp with the *E. coli* consensus sequence (TGTGA-N_6_-TCACA; Figure 3B). Electrophoretic mobility shift assays (EMSA) were performed using a recombinant His_6_-CRP protein and DNA probes. Indeed, CRP bound to the *aroX*-*npsA* intergenic region since CRP-DNA complexes were observed using 100nM of His_6_-CRP and this DNA-binding activity was dependent of the presence of cAMP (200μM; Figure 3C). To demonstrate whether the putative CRP-box was required for CRP binding to the *aroX*-*npsA* intergenic region, site-directed mutagenesis of the CRP-box was performed (Figure 3B). CRP did not bind to the *aroX*-*npsA* intergenic region containing the mutation of CRP-Box (Figure 3D). As positive and negative controls, *estA2* and *eltA* regulatory regions were used (Figures 3E,F; Haycocks et al., 2015). These results clearly show that CRP binds directly to the *aroX*-*npsA* intergenic region through recognition of a specific binding site.

### CRP Induces TV-Mediated Cytotoxicity on Epithelial Cells

**Figure 4:**
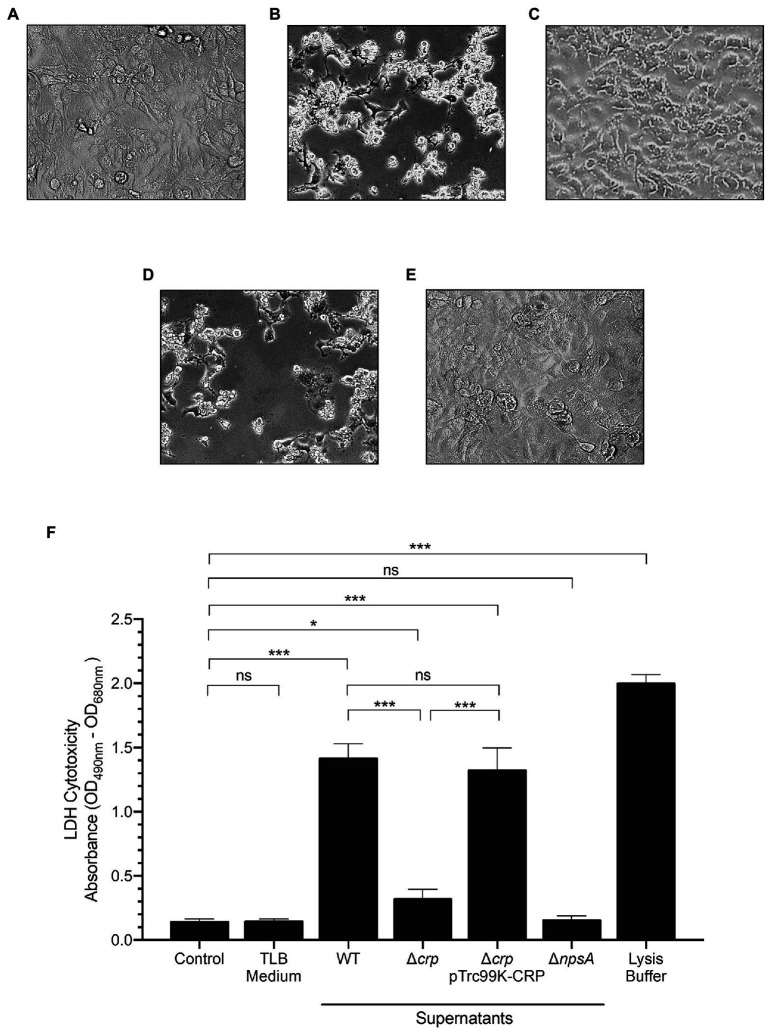
Cytotoxicity of *K. oxytoca* Δ*crp* mutant strain. HeLa cell culture inoculated with: **(A)** TLB medium as control, and **(B)** wild-type **(C)** Δ*crp* **(D)** Δ*crp* pTrc99K-CRP, and **(E)** Δ*npsA K. oxytoca* supernatants. **(F)** HeLa cells were treated with TLB medium, and *K. oxytoca* supernatants (wild-type, Δ*crp*, Δ*crp* pTrc99K-CRP, and Δ*npsA*) for 48h. Following treatment, aliquots were obtained for measurement of extracellular LDH. Minimal and maximal measurable LDH release was determined by incubating cells with PBS (control), and lysis buffer, respectively. Statistically significant: ^*^p<0.05; ^***^p<0.001; ns: not significant. All values of *p* were calculated using one-way ANOVA and Tukey’s comparison test.

To corroborate the role of CRP on the *aroX* and *npsA* genes transcription, we determined the TV-mediated cytotoxicity on epithelial cells using the supernatants from *K. oxytoca* wild type, ∆*crp* mutant, and complemented ∆*crp* mutant. Cytotoxicity was severely affected in the ∆*crp* mutant as compared to the wild type and the complemented mutant. As expected, the supernatant of the non-toxigenic ∆*npsA* strain did not cause any cytotoxic effect on HeLa cells (Figure 4A). Further, the LDH release activity assay showed that wild-type strain supernatant induced death of HeLa cells (~14-fold) as compared to the PBS control. In contrast, the ∆*crp* mutant supernatant significantly reduced the death of HeLa cells by ~6-fold as compared to the wild-type strain supernatant. Levels of released LDH by HeLa cells treated with the supernatant of the complemented ∆*crp* mutant were similar to that of wild-type strain supernatant. Neither the TLB medium nor the non-toxigenic ∆*npsA* supernatant induced death of HeLa cells (Figure 4B). These phenotypic data support the role of CRP global regulator as a transcriptional activator of genes involved in the *K. oxytoca* TV biosynthesis.

## Discussion

*K. oxytoca* is a pathobiont of the intestinal microbiota that can produce TV cytotoxin (Beaugerie et al., 2003; Högenauer et al., 2006; Zollner-Schwetz et al., 2015; Glabonjat et al., 2021). After penicillin treatment, alteration of the enteric microbiota occurs, and overgrowth of *K. oxytoca* is originated in the colon causing a severe dysbiosis (Schneditz et al., 2014; Hajishengallis and Lamont, 2016; Dornisch et al., 2017; von Tesmar et al., 2018; Alexander et al., 2020). The imbalance in the gut microbiota and the production of TV cytotoxin result in AAHC (Högenauer et al., 2006; Unterhauser et al., 2019). The *aroX* and NRPS operons encode the proteins involved in the biosynthesis of TV and are clustered in a PAI that is only present in the *K. oxytoca* toxigenic strains (Schneditz et al., 2014; Dornisch et al., 2017). In this study, we analyzed the expression of *aroX* and NRPS operons in strains growing in various culture media since previous work has determined that nutritional components are environmental stimuli that trigger a differential expression of genes related to bacterial virulence (Blair et al., 2013; De la Cruz et al., 2017; Han et al., 2017; Ares et al., 2019; Soria-Bustos et al., 2020; Jiang et al., 2021). Our results showed that TLB culture medium significantly enhanced the expression of the *aroX* and NRPS operons, in agreement with a previous report in which the production of TV significantly increased in the toxigenic strain *K. oxytoca* MH43-1 when the bacterium was grown in TLB as compared with TSB and LB (Tse et al., 2017). A previous report showed that TV production reaches maximum levels at late exponential and stationary growth phases (Joainig et al., 2010). We determined the expression of *aroX* and *npsA* genes during 12h of growth in TLB medium and, as expected, transcription attained maximum levels at 9h and remained as such until 12h of growth, which corresponds to stationary growth phase. In addition to the effect of nutritional components from different culture media being analyzed, we evaluated the influence of other environmental stimuli such as glucose, glycerol, osmolarity, and divalent cations. Regarding the role of carbon source in transcription, glucose and glycerol repressed and activated the *aroX* and *npsA* gene expression, respectively. It was reported that glucose and glycerol have antagonistic activities in the control of cAMP production, which is necessary for the regulatory activity of the CRP protein (Soberón-Chávez et al., 2017). While glucose inhibits, glycerol induces cAMP synthesis (Deutscher et al., 2006; Fic et al., 2009; Pannuri et al., 2016). Glucose concentration is higher in the small intestine, while glycerol is produced abundantly by enteric microorganisms residing in the colon (Yuasa et al., 2003; Fujimoto et al., 2006; Ohta et al., 2006; De Weirdt et al., 2010). Our results suggest that the genes involved in TV biosynthesis could be expressed in the glycerol-rich colon environment under regulation of CRP. We found here that sodium chloride repressed *aroX* and *npsA* gene expression. Previously, it was reported that when *Listeria monocytogenes* grows in the presence of high concentrations of sodium chloride, some genes that code for metabolic enzymes and virulence factors are repressed (Bae et al., 2012). In enterotoxigenic *E. coli,* the addition of sodium chloride to culture medium also repressed transcription of the coli surface antigen CS3 (Ares et al., 2019). In contrast, high osmolarity increases the gene expression of the enterotoxigenic *E. coli* Longus pilus (De la Cruz et al., 2017). The ubiquitous divalent cations magnesium and calcium are important nutrients required by bacteria for growth and cell maintenance. The effects of calcium and magnesium can be highlighted in physio-chemical interactions, gene regulation, and bio-macromolecular structural modification (Groisman et al., 2013; Wang et al., 2019). Moreover, it has been reported that divalent cations are involved in transcriptional regulation of virulence factors in pathogenic *E. coli*, *Aeromonas hydrophila*, *P. aeruginosa*, *Vibrio cholerae,* and *Yersinia enterocolitica* (Fälker et al., 2006; Bilecen and Yildiz, 2009; Vilches et al., 2009; Anderson et al., 2010; De la Cruz et al., 2017; Ares et al., 2019; Liu et al., 2020c). In our study, the presence of calcium and magnesium divalent cations was required for *aroX* and *npsA* gene expression.

This work underlines for the first time, the regulatory activity of CRP in the transcription of genes involved in TV biosynthesis. The *K. oxytoca* CRP protein is homologous to other CRP proteins from several enteropathogens such as *K. pneumoniae*, *E. coli*, and *S. enterica*. The absence of CRP exhibited a growth defect in both exponential and stationary phases, as observed in *Vibrio vulnificus* (Kim et al., 2013), *E. coli* (Basak et al., 2014), *K. pneumoniae* (Ou et al., 2017; Lin et al., 2018), and *Haemophilus parasuis* (Jiang et al., 2020). These observations are in accordance with previous reports that explain how growth fitness is affected by the *crp* deletion, which produces fluctuations of metabolic gene expression and alterations of carbon metabolites, α-ketoacids, cAMP, and amino acids that promote proper coordination of protein biosynthesis machinery during metabolism (Klumpp et al., 2009; Berthoumieux et al., 2013; You et al., 2013; Pal et al., 2020). CRP is a central regulator of carbon metabolism and has been implicated as an important facilitator of host colonization and virulence in many bacterial pathogens, including *K. pneumoniae* (Xue et al., 2016; Lin et al., 2018; Panjaitan et al., 2019). In this study, we demonstrated that CRP positively regulates the expression of the *aroX* and NRPS operons and plays a regulatory role in response to lactose as was previously demonstrated in *E. coli* and *K. pneumoniae*, where lactose acts as glycerol inducing the augmentation of cAMP (Deutscher et al., 2006; Panjaitan et al., 2019). We also showed that CRP directly activates the expression of *aroX* and *npsA* genes by binding to their intergenic regulatory region and that cAMP is indispensable for this DNA-binding activity.

Cytotoxic effects of toxigenic *K. oxytoca* strains have been previously reported (Minami et al., 1989, 1994; Higaki et al., 1990; Beaugerie et al., 2003; Darby et al., 2014; Schneditz et al., 2014; Tse et al., 2017; Paveglio et al., 2020). We found that the Δ*crp* mutant strain was remarkably less cytotoxic than the wild-type strain. This was due to downregulation of the TV biosynthetic *aroX* and NRPS operons and reduced TV production in the absence of CRP.

## Conclusion

This study underscores the role of the CRP-cAMP signaling pathway in the activation of genes involved in TV biosynthesis of toxigenic *K. oxytoca* and provides clues about the intestinal signals that trigger CRP-mediated TV production.

## Data Availability Statement

The original contributions presented in the study are included in the article/Supplementary Material, and further inquiries can be directed to the corresponding authors.

## Author Contributions

DR-V, MC, and MA conceived and designed the study. DR-V, NL-M, JS-B, JM-C, RG-U, RR-R, and LG-M performed the experiments. DR-V, SR-G, JG-M, HH, JF, MC, and MA analyzed the data. DR-V, JAG, MC, and MA wrote the manuscript. All authors contributed to the article and approved the submitted version.

## Conflict of Interest

The authors declare that the research was conducted in the absence of any commercial or financial relationships that could be construed as a potential conflict of interest.

## Publisher’s Note

All claims expressed in this article are solely those of the authors and do not necessarily represent those of their affiliated organizations, or those of the publisher, the editors and the reviewers. Any product that may be evaluated in this article, or claim that may be made by its manufacturer, is not guaranteed or endorsed by the publisher.

## Footnotes

^1^ http://primer3plus.com/cgi-bin/dev/primer3plus.cgi

## Supplementary Material

Supplementary Figure S1 Expression of reference gene (*rrsH*) under the different conditions tested in this study. Panels show the expression of reference gene in different: **(A)** growth conditions, **(B)** growth phases, **(C)** environmental cues, **(D)** *K. oxytoca* strains, and **(E)** growth culture medium with or without lactose. Quantification of expression is showed as *rrsH* (16S rRNA) copies per microgram of RNA.
