# Supplementary figures and images for: cAMP Receptor Protein Positively Regulates the Expression of Genes Involved in the Biosynthesis of Klebsiella oxytoca Tilivalline Cytotoxin
Source: Front Microbiol. 2021 Sep 30;12:743594. doi: 10.3389/fmicb.2021.743594 (PMC8515920; doi:10.3389/fmicb.2021.743594)

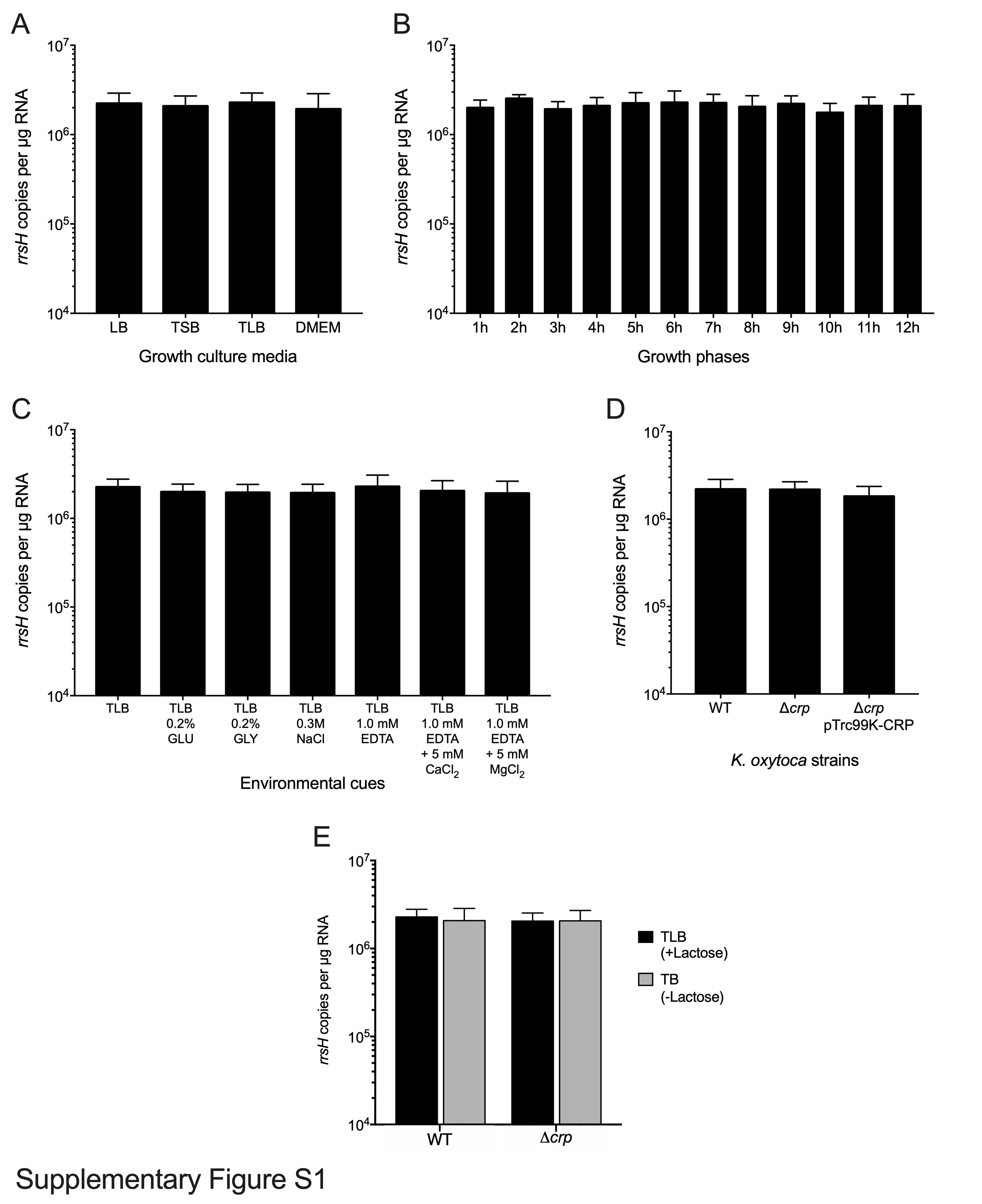

Supplement: Supplementary Figure S1 — Expression of reference gene (rrsH) under the different conditions tested in this study. Panels show the expression of reference gene in different: (A) growth conditions, (B) growth phases, (C) environmental cues, (D) K. oxytoca strains, and (E) growth culture medium with or without lactose. Quantification of expression is showed as rrsH (16S rRNA) copies per microgram of RNA. [file Image_1.TIFF]
